# Phytochemical activity and antioxidant activity of hidroethanolic extract and fractions from *Poincianella pyramidalis*(tul.) L.P.Queiroz var. *pyramidalis*stembark

**DOI:** 10.1186/1753-6561-8-S4-P257

**Published:** 2014-10-01

**Authors:** Sabrina Zelice da Cruz de Moraes, Andre Luiz Lima Menezes dos Santos, Ludmila Cruz dos Santos, Antonio Santos Dias, Aline Camila Silva de Oliveira, Charles Santos Estevan, Jose Davi Prado Lima, Andrea Yu Kwan Villar Shan

**Affiliations:** 1 Physiology Department, Sergipe Federal University, São Cristóvão, SE, Brazil

## Background

Plants are an important source of bioactive compounds with different chemical compositions and biological properties with proven efficacy. *Poincianell pyramidalis *(*Poincianella pyramidalis *(Tul.) LP. Queiroz var. *pyramidalis*), popularly known as catingueira, is a medicinal plant endemic to the Caatinga and almost all parts are used for the treatment of many diseases, such as asthma, gastritis, fever, diarrhea, stomach pains and coughing. Despite the great biodiversity in Brazil and the therapeutic potential presented by several species, there are few studies in vivo and in vitro of extracts derived from plants. Thus, the general objective of this study was to outline the phytochemical profile, evaluate the antioxidant activity of extract and fractions and analyze the chromatographic profile by HPLC-DAD from polar fractions of the stem bark of *P. pyramidalis*.

## Methods

The hydroethanolic extract was obtained by maceration with 90% ethanol and hexane fractions (FHX), chloroform (FCL), ethyl acetate (EAF) and hydromethanol (FHM) were obtained by liquid-liquid partition. Antioxidant activity was measured using the scavenging of free radical DPPH (2,2-diphenyl-1-picrildridrazila) [2,3] and TBARS (reaction of thiobarbituric acid metabolites) [3]. Data were expressed as percentage inhibition (PI) for DPPH and TBARS and antioxidant activity index (AAI) and EC50 (50% effective concentration) for DPPH. The EC50 was calculated from linear and non-linear regression. Total phenolic compounds quantification was performed by Folin Ciocautel [2], and high performance liquid chromatography (HPLC-DAD) was used in order to assume the chemical composition of EAF fraction. Data were presented as mean ± standard deviation and differences between groups were determined by ANOVA followed by Tukey test (p <0,05).

## Results and conclusion

Among all samples, the FAE was the one with the highest percentage of inhibition for the two models, 90% and 50% respectively. AAI of this fraction (23,8) is considered very strong (AAI>2,0) [4], EC50 fraction had no significant difference compared to gallic acid (p <0.05). This fraction also showed higher levels of total phenols, 3.65 ± 1.00 mg GAE / g. The chromatogram and UV spectrum of the major peaks retention time of 30.83 min and 34.59 min suggest the presence flavones in FAE, but further tests are required to investigate whether the effects of samples are related to this compound alone or the synergism of flavone with other secondary metabolites. It can be concluded that the extract and fractions from *Poincianella pyramidalis *have good antioxidant effect, which is related to the quantity of secondary metabolites present in the crude extract and its fractions, which underpins scientifically the medicinal use of this plant by the population.
